# The Adverse Events of Oxycodone in Cancer-Related Pain: A Systematic Review and Meta-Analysis of Randomized Controlled Trials

**DOI:** 10.1097/MD.0000000000003341

**Published:** 2016-04-18

**Authors:** Hu Ma, Yuan Liu, Lang Huang, Xian-Tao Zeng, Su-Han Jin, Guo-Jun Yue, Xu Tian, Jian-Guo Zhou

**Affiliations:** From the Department of Oncology, Affiliated Hospital of Zunyi Medical University (HM, LH, G-JY, J-GZ); Center for Translational Medicine (HM, LH, J-GZ) and Department of Pharmacology and Key Laboratory of Basic Pharmacology of Ministry of Education (YL), Zunyi Medical University; Department of Cardiology and Endodontics, Affiliated Stomatological Hospital of Zunyi Medical University (S-HJ), Zunyi; Center for Evidence-Based and Translational Medicine, Zhongnan Hospital of Wuhan University (X-TZ); Center for Evidence-Based and Translational Medicine, Wuhan University (X-TZ), Wuhan; Graduate College and School of Nursing, Tianjin University of Traditional Chinese Medicine, Tianjin (XT), China.

## Abstract

The adverse events (AEs) of oxycodone in cancer-related pain were controversial, so we conducted a meta-analysis to determine it.

PubMed, Embase, CBM, CNKI, WanFang database, The Cochrane library, Web of Science, and the reference of included studies were searched to recognize pertinent studies. Relative risk (RR) with 95% confidence intervals (CIs) for all AEs were all extracted. The fixed-effects model was used to calculate pooled RRs and 95% CIs. Power calculation was performed using macro embedded in SAS software after all syntheses were completed.

We identified 11 eligible trials involving 1211 patients: 604 patients included in oxycodone group and 607 patients involved in control group. Our quantitative analysis included 8 AEs, and the pooled analyses indicated that oxycodone compared with other opioids in cancer-related pain were not significantly decreased RRs of all AEs (dizziness RR = 0.94, 95% CI: 0.69–1.30, Z = 0.35, *P* = 0.72; nausea RR = 0.88, 95% CI: 0.72–1.07, Z = 1.26, *P* = 0.21; vomiting RR = 0.89, 95% CI: 0.70–1.15, Z = 0.9, *P* = 0.37; sleepiness RR = 0.86, 95% CI: 0.38–1.36, Z = 0.36, *P* = 0.72; constipation RR = 0.98, 95% CI: 0.81–1.19, Z = 0.21, *P* = 0.83; anorexia RR = 0.97, 95% CI = 0.58–1.62, Z = 0.11, *P* = 0.91; pruritus RR = 0.76, 95% CI: 0.44–1.30, Z = 1.01, *P* = 0.31; dysuria RR = 0.33, 95% CI: 0.07–1.62, Z = 1.36, *P* = 0.1)]. The subgroup analysis shown that Ox controlled-release (CR) had less sleepiness compared with MS-contin (Mc) CR (RR = 0.47, 95% CI: 0.25–0.90, *P* = 0.02). The power analysis suggests that all AEs have low statistical power.

The present meta-analysis detected that no statistically significant difference were found among oxycodone and other opioids in all AEs, but Ox CR may had less sleepiness compared with Mc CR when subgroup analysis were conducted.

## INTRODUCTION

Pain is 1 of the most common symptoms in cancer patients. Approximately 60% patients experience pain, one-third of the patients who graded their pain as moderate or severe.^1^ Continued pain related to less interaction with family and friends, much less motivation, and poor quality of life. The guideline of treatment for cancer patients with pain was issued by the World Health Organization (WHO), which was the principle was the foundation for the treatment of cancer-related pain. However, WHO was not suitable at present. Now new opioids guidelines^2^ was published with overcoming some limitations, and was accepted by most oncologists. In the past, opioids have been used in cancer patients who experience moderate and severe pain many years. Oxycodone, 1 of opioids, has been used in clinic since 1917, and a series of randomized controlled trials (RCTs) about oxycodone used in advanced cancer patients with pain.^3–10^ Although many meta-analyses and systematic review published,^1,11–15^ and most of meta-analyses focused on efficacy of this agent, but few of them confirmed the side effects. Wang et al^12^ detected that oxycodone significantly decreased the incidence of nausea and constipation compared with other opioids, while recently studies^3–5^ found that the adverse events (AEs) were similar among oxycodone and other opioids. Oosten et al^14^ reviewed the common AEs of opioids for cancer-related pain, but the review did not pooled analysis the side effects, the descriptive analyses could not be observed visually by anyone.

Therefore, a meta-analysis and power analysis was carried out to compare the all AEs among oxycodone and other opioids in the management of cancer-related pain based on currently available studies.

## METHODS

Ethical approval and patient written informed consent are not necessary because of this is not primary research. This study was conducted following the Preferred Reporting Items for Systematic Reviews and Meta-Analyses (PRISMA) statement (the detail of PRISMA was presented in Supplemental Data 1),^16^ and according to *Cochrane Handbook for Systematic Reviews of Interventions*. The protocol was registered by Centre for Reviews and Dissemination PROSPERO (available at: http://www.crd.york.ac.uk/prospero/register_new_review.asp?RecordID=13401&UserID=7339) (Registration No. CRD 42014013401).

### Search Strategy

Eligible trials were identified through electronically searching the databases of PubMed, Web of Science, The Cochrane library, and EMBASE, China National Knowledge Infrastructure (CNKI), WanFan Database, China Biomedical Literature database (CBM), and Chinese Science and Technology Periodical Database (VIP) using the following terms: (“Pain Measurement” OR “Pain”) AND (“Tumors” OR “Cancer” OR “Neoplasms”) AND (“Oxycodone” OR “Oxycone” OR “Dinarkon”) (from inception to November 28, 2015, update in January 22, 2016). The search strategy for PubMed and Embase were summarized in Supplemental Data 2. Language or date restrictions were not imposed. We manually checked the bibliographies of previous reviews and included trials to identify other potentially eligible trials.

### Selection Criteria

All studies focused on the all AEs among oxycodone and other opioids in cancer-related pain were involved by using following selection criteria—Population: patients were diagnosed as cancer, with no other restrictions; Intervention: oxycodone plus other agents or alone, regardless of any formulation and any route of administration; Comparison: other opioids (e.g., morphine, oxymorphone), regardless of extended-release or other formulation; Outcomes: all of the side effects will be evaluated; Study design: RCTs, no matter parallel-or cross-over group.

### Data Extraction

Two reviewers (HM and LH) independently screened the titles and abstracts to exclude studies that were not reach the inclusion criteria, then the full-text articles were read. Finally, data extraction was conducted using a premade data extraction form based on electronic database to collect information as following: authors, the population studied, publication year, country, the formulation of oxymorphone or control, and the detailed information regarding Patient(P), Intervention(I), Comparison(C) and Outcome(O) study design(s) (PICOs). Extracted data were entered into a database, which created by EpiData version 3.1.

### Assessment for Risk of Bias

Two reviewers (HM and YL) independently evaluated the risk of bias using the Cochrane Collaboration tool.^17^ The authors estimated the following domains: random sequence generation, allocation concealment, blinding of participants and personnel, blinding of outcome assessment, incomplete outcome data, selective reporting, and other bias. Based on the information extracted from included studies, each domain was assigned as a value of “high risk,” “unclear risk,” or “low risk.” Any disagreement between searchers concerning the eligibility of a trial was resolved by consulting a third reviewer (J-GZ).

### Grading Quality of Evidence

Two authors (J-GZ and XT) independently evaluated the quality of evidence for all of AEs following the GRADE methodology for risk of bias, inconsistency, indirectness, imprecision, and publication bias; assigned as very low, low, moderate, or high. The summary table of quality of evidence was made using GRADE Profiler (GRADEpro, version 3.6) (available at: http://www.gradeworkinggroup.org/).^18^

### Statistical Analysis

Except for publish bias used STATA version 12.0 (Stata Corp, College Station, TX), all analysis used RevMan (Version 5.3. Copenhagen: The Nordic Cochrane Centre, The Cochrane Collaboration, 2014) in this meta-analysis. We estimated the relative risk (RR) with 95% confidence intervals (CIs) for dichotomous outcomes. I^2^ statistic and *P* value were used to estimate the level of heterogeneity of included studies.^19^ We considered heterogeneity substantial if I^2^ ≥ 50% or *P* < 0.10.^20^ On the contrary, if obvious difference were found in clinical characteristic and/or methodology, regardless of I^2^ statistic or *P* value, a belief of qualitative analysis was conducted.^19^ We also apply subgroup analysis for all AEs according to 5 arms (Ox CR vs Mc CR, Ox CR vs Mo CR, Ox PR vs Oxn PR, Ox CR vs Omo CR, or Ox CR vs Dc CR). The presence of publication bias was evaluated by using Begg and Egger regressions.^21,22^ We considered a *P* value of <0.05 to be statistically significant.^23^

### Power Analysis

Power calculation was performed using the methodology described by Cafri et al^23,24^ after all syntheses were performed by SAS version 9.21 (SAS Institute Inc., Cary, NC). Details on the macro and SAS code used were included in the online supplement.

## RESULTS

### Literature Research and Characteristic of Studies

**FIGURE 1:**
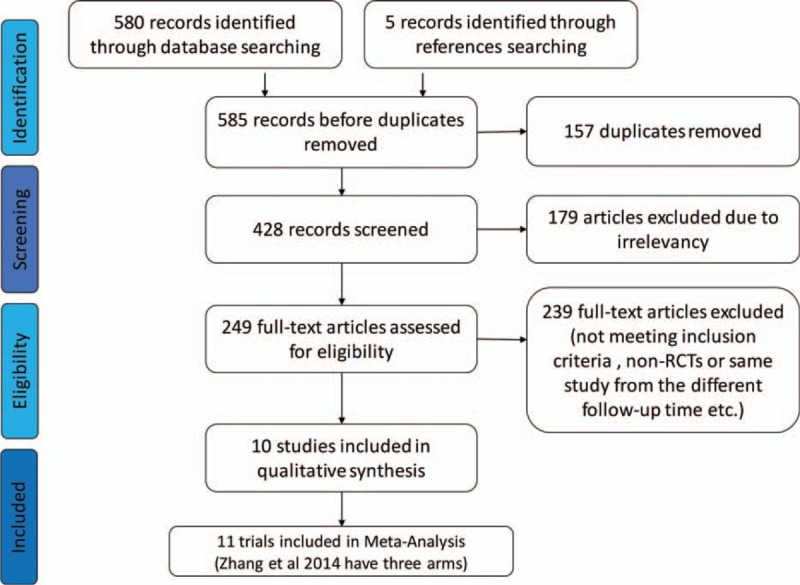
Flow diagram of the details of the study.

A total of 580 unfiled titles and abstracts were identified through database searching and 5 records identified through references searching. Finally 10 studies^3–10,25,26^ with 11 trials and 1211 patients were involved in this meta-analysis, and 604 patients included in oxycodone group and 607 patients involved in control group, respectively, and the sample size ranged from 30 to 248. Moreover, 126 patients with cancer-related pain have appeared dizziness, 269 patients have appeared nausea, 188 patients have occurred vomiting, 60 patients with cancer pain have occurred sleepiness, 42 patients have reported pruritus, 260 patients have reported constipation, 49 patients shown anorexia, and 6 patients have reported dysuria, and all side effects being included in the final analysis. The flow diagram of the literature searched and evaluated was presented in Figure 1.

All eligible studies were published between 2002 and 2015. In total, 10 studies provided outcomes, the trial finished by Zhang et al^4^ was an RCT with 3-arm design comparing morphine, MS contin and oxycodone in treatment of cancer pain. Nausea, vomiting, and constipation were available in all of trials, dizziness was appeared in 10 trials, anorexia and pruritus were reported in 4 trials, sleepiness was occurred in 5 trials, dysuria was reported in 3 trials, and insolence was appeared in 2 trials. Riley et al^3^ reported that opioid adverse reaction scores were scored on an 11-point Numerical Rating Scale, Yu et al^5^ shown that treatment-emergent adverse events (TEAEs) were reported by either patients or interviewers, Heiskanen and Kalso^26^ used Modified Specific Drug Effect Questionnaire to assessment AEs, Mucci-LoRusso et al^10^ used the Specific Drug Effect Questionnaire to evaluation side effects, Gabrail et al.^8^ AEs were rated by investigators; however, the other studies did not report the method to assessment TEAE. Five studies come from China,^4,5,7,9,25^ 2 come from United States,^8,10^ the others come from United Kingdom,^3^ Finland,^26^ and Europe.^6^ Five trials compared oxycodone (Ox) controlled-release (CR) with MS-contin (Mc) CR,^3–5,7,25^ 3 trials compared Ox CR with morphone (Mo) CR,^4,10,26^ the other trials were Ox prolonged-release (PR) vs oxycodone/naloxone (Oxn) PR,^6^ Ox CR vs oxymorphone (Omo) CR,^8^ and Ox CR vs DHC-contin (Dc) CR,^9^ respectively. The main characteristics of the included studies were recorded in Table 1.

**TABLE 1:**
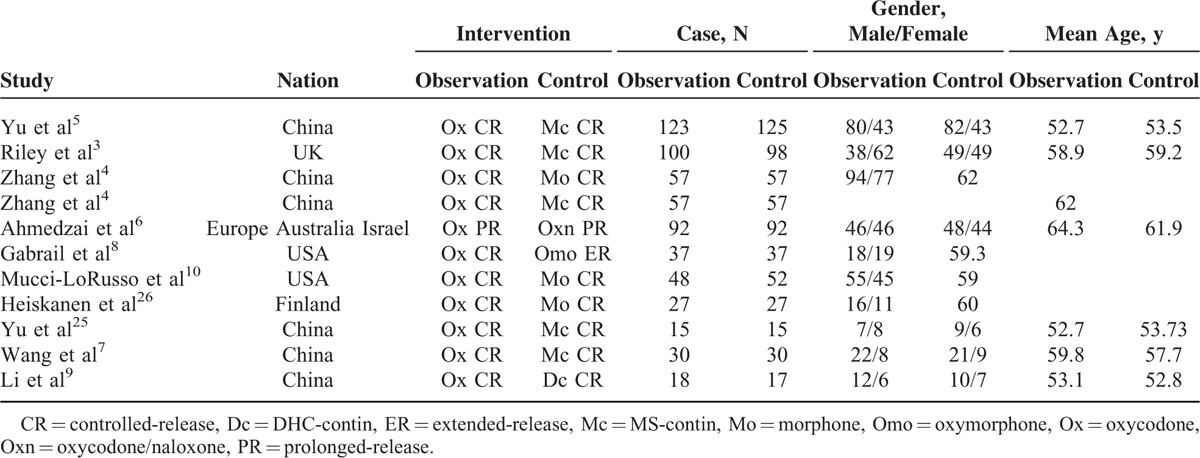
Main Characteristics of the Studies

### Assessing Risk of Bias

**FIGURE 2:**
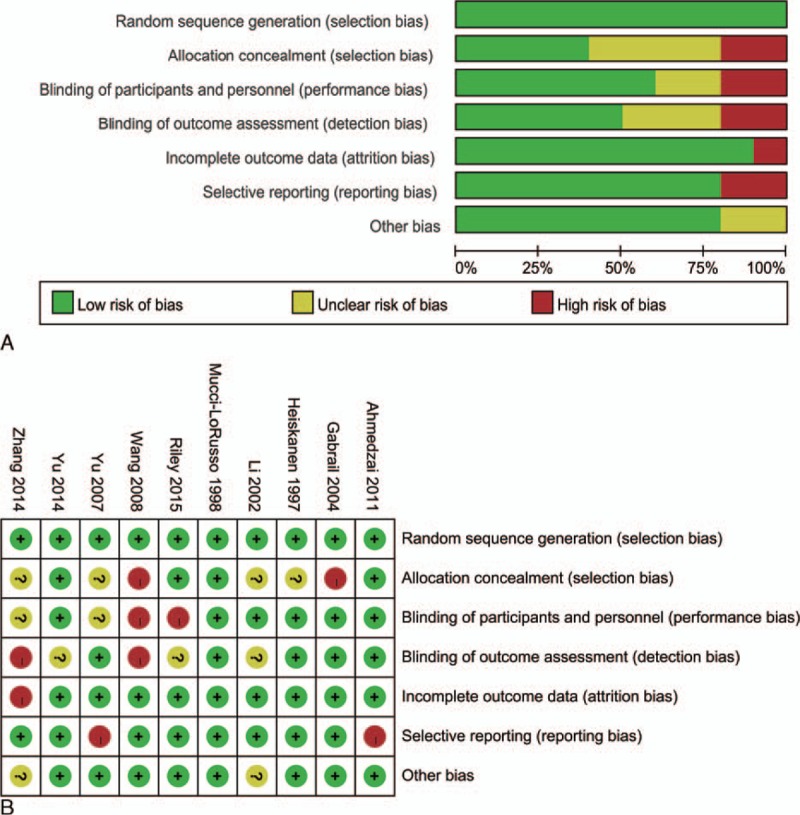
Appraisal of risk of bias of the included trials using the Cochrane risk-of-bias tool. Low risk = bias, if present, is unlikely to alter the results seriously, unclear risk = bias raises some doubt about the results, high risk = bias may alter the results seriously.

The detail of the risk-of-bias assessment was summarized in Figure 2. Ten eligible studies were incorporated into our meta-analysis. All studies generated an adequate randomization sequence, but only 1 study shown the detail of randomization.^3^ Two of all the studies were assessed as high risk in allocation concealment, 4 as unclear risk, and others as low risk of bias. Two studies have blinding of participants, personnel, and outcome assessment. Only 1 study possessed of incomplete outcome data, and 8 of all eligible studies reported selective reporting. Nevertheless, there are some criteria of assessments judged as high bias; however, those unlikely to affect the quality assessment. The overall methodological quality was generally good and fair.

### RR of All AEs

There are 8 AEs including in this systematic review to quantitative analysis, and sleepiness found significant heterogeneity (I^2^ = 56%, *P* = 0.06), so a random model was used. The rest of AEs calculated results are I^2^ < 50% and *P* > 0.10, and did not detect significant heterogeneity, so we choose the fixed model to meta-analysis about them. We found no significant difference in the RRs of overall AEs. The results of subgroup analysis are following.

#### Dizziness

**FIGURE 3:**
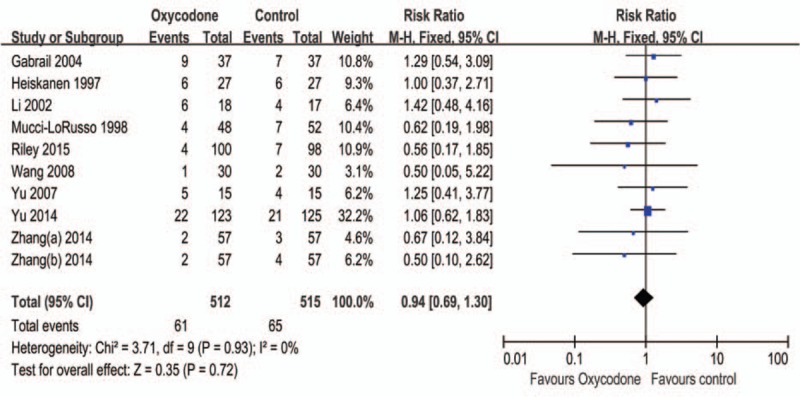
Meta-analysis result of the relative risk of dizziness.

Ten RCTs reported the dizziness events, 5 RCTs were Ox CR vs Mc CR, and 3 RCTs were Ox CR vs Mo CR, others were Ox CR vs Omo CR and Ox CR vs Dc CR. As is shown in Figure 3, the overall pooled RR of dizziness is 0.94 (95% CI: 0.69–1.30, Z = 0.35, *P* = 0.72), the subgroup analysis shown that no statistical difference between differently control groups (Table 2).

**TABLE 2:**
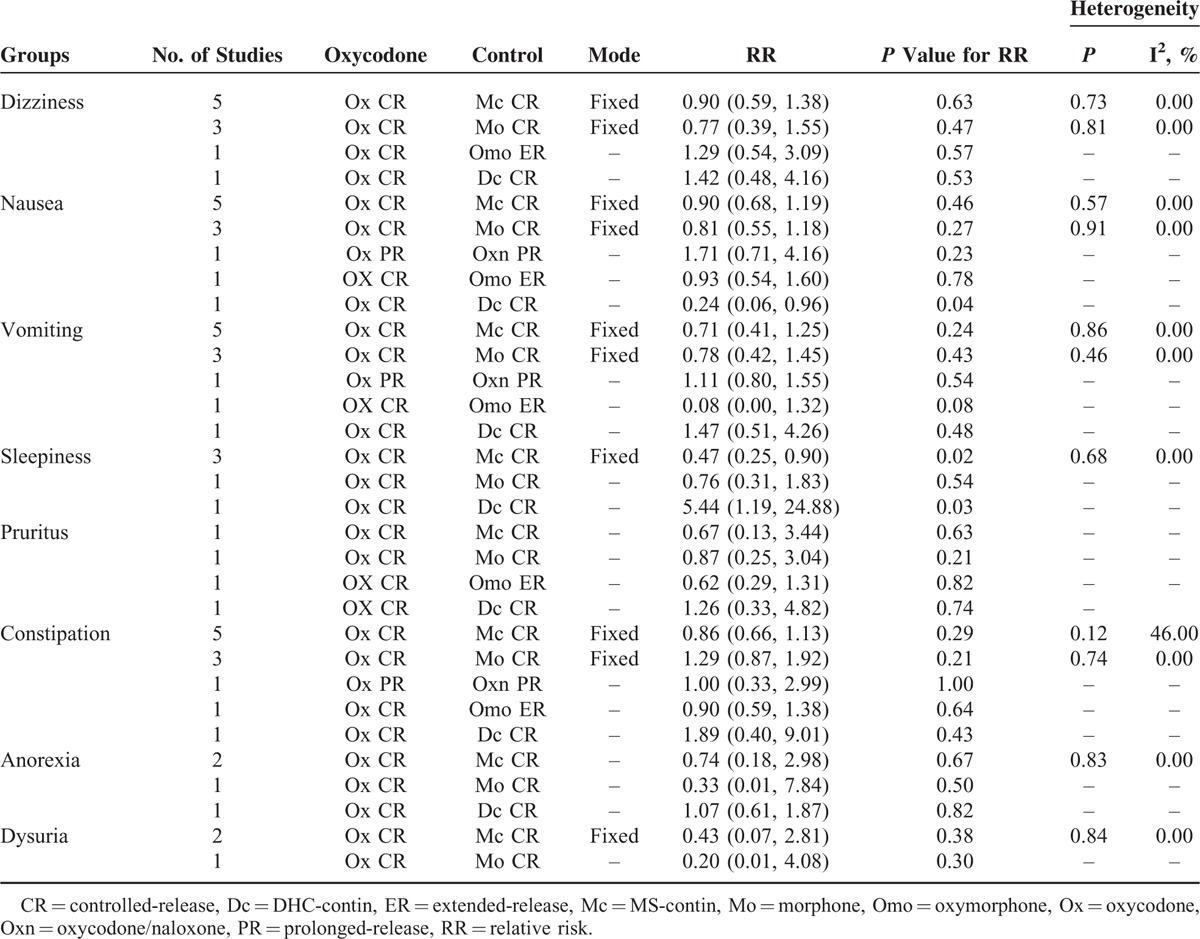
Subgroup Analysis Among Ox CR and Other Dosage Form Opioids in Cancer-Related Pain

#### Nausea

**FIGURE 4:**
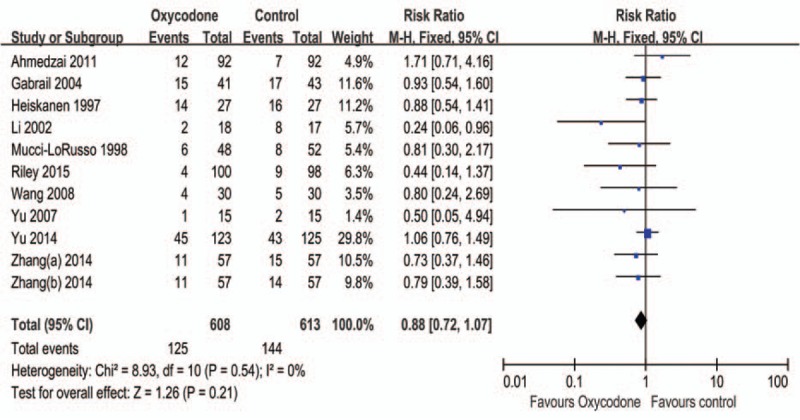
Meta-analysis result of the relative risk of nausea.

This meta-analysis of nausea including 11 trials, 5 RCTs were Ox CR vs Mc CR, and 3 RCTs were Ox CR vs Mo CR, others were Ox PR vs Oxn PR, Ox CR vs Omo CR, and Ox CR vs Dc CR. The result did not detect statistically significant difference, and the pooled RR is 0.88 (95% CI: 0.72–1.07, Z = 1.26, *P* = 0.21) (Figure 4), besides the subgroup analysis did not change the result (Table 2).

#### Vomiting

**FIGURE 5:**
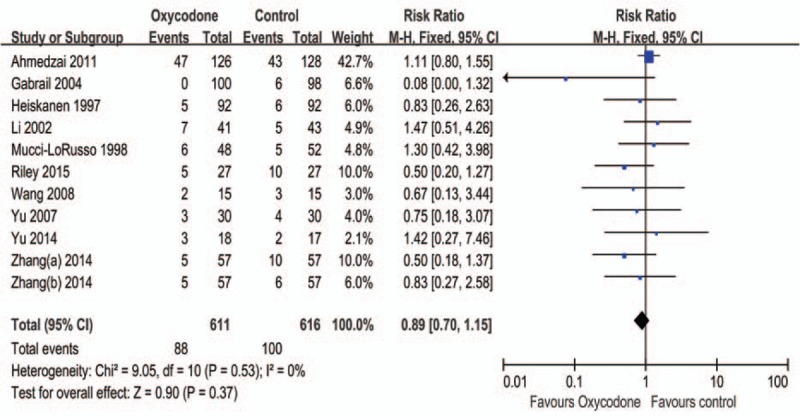
Meta-analysis result of the relative risk of vomiting.

All studies bring into this meta-analysis, the intervention arms and controls are shown in Table 1. Figure 5 suggests that oxycodone is not statistically different between control groups, the pooled RR is 0.89 (95% CI: 0.70–1.15, Z = 0.9, *P* = 0.37), and the subgroup analysis did not detect superiority of different agents (Table 2).

#### Sleepiness

**FIGURE 6:**
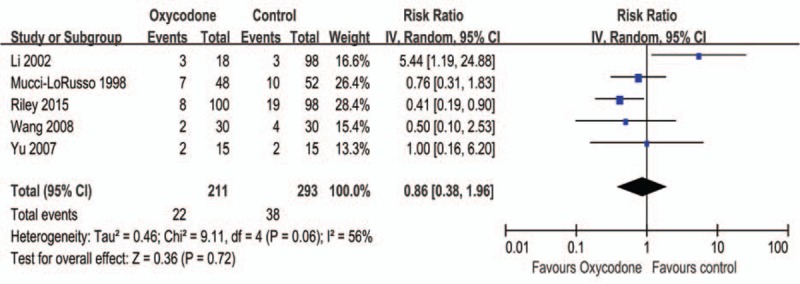
Meta-analysis result of the relative risk of sleepiness.

Five trials including in meta-analysis with random model, 211 participates are received oxycodone and 212 participates with other agents. The result suggest that oxycodone did not decrease the risk of sleepiness (RR = 0.86, 95% CI: 0.38–1.96, Z = 0.36, *P* = 0.72; Figure 6). The subgroup analysis shown that Ox CR had less sleepiness compared Mc CR (RR = 0.47, 95% CI: 0.25–0.90, *P* = 0.02), while Mo CR or Dc CR compared with Ox CR had no statistical difference, respectively (Table 2).

#### Constipation

**FIGURE 7:**
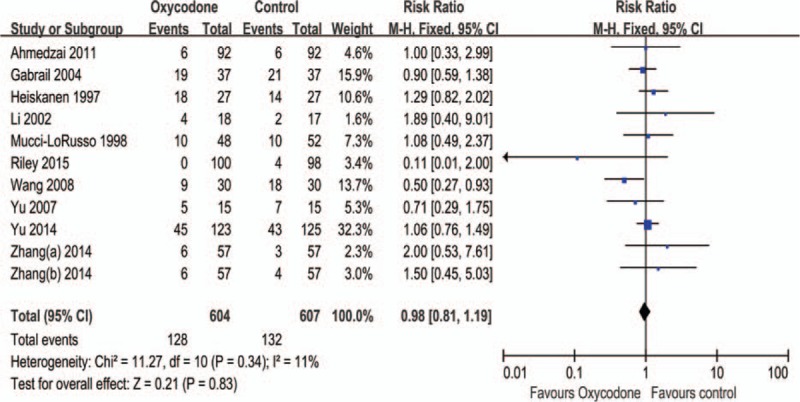
Meta-analysis result of the relative risk of constipation.

All of RCTs included in this meta-analysis, we found no significant difference in constipation, the pooled RR is 0.98 (95% CI: 0.81–1.19, Z = 0.21, *P* = 0.83; Figure 7), the subgroup analysis did not detect difference of all comparisons (Table 2).

#### Anorexia

**FIGURE 8:**
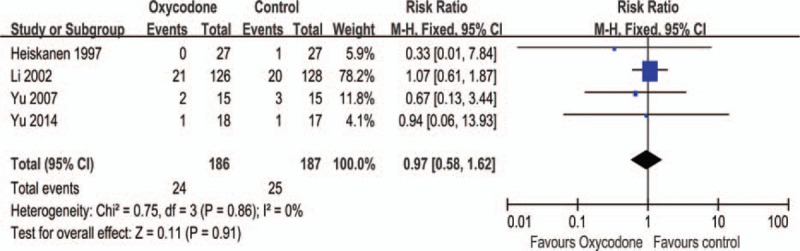
Meta-analysis result of the relative risk of anorexia.

Four studies^5,9,25,26^ reported anorexia, we found no significant difference of RR (RR = 0.97, 95% CI: 0.58–1.62, Z = 0.11, *P* = 0.91; Figure 8), the subgroup analysis shown no difference of Mc CR, Mo CR, or Dc CR (Table 2).

#### Pruritus

**FIGURE 9:**
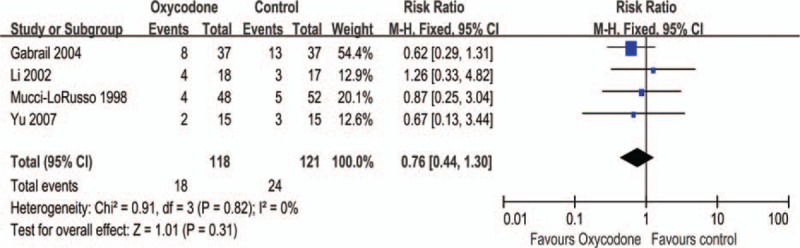
Meta-analysis result of the relative risk of pruritus.

The side effect was reported in 4 studies,^8–10,25^ the pooled analysis indicated that no significant difference in incidence of pruritus (RR = 0.76, 95% CI: 0.44–1.30, Z = 1.01, *P* = 0.31; Figure 9). No significant difference was detected by the subgroup analysis (Table 2).

#### Dysuria

**FIGURE 10:**
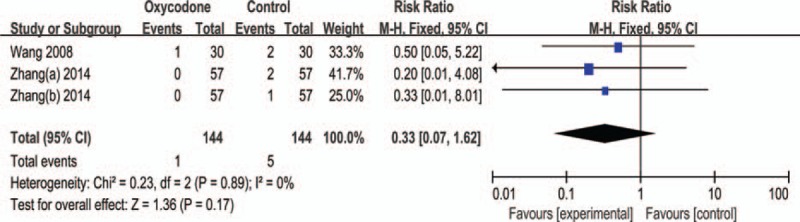
Meta-analysis result of the relative risk of dysuria.

Three trails^4,7^ reported dysuria, we found no significant difference of dysuria (RR = 0.33, 95% CI: 0.07–1.62, Z = 1.36, *P* = 0.17; Figure 10). We did not find any difference between the subgroup (Table 2).

### Power Analysis

Power calculations were conducted post hoc after all the analyses had been completed by using the methodology described by Cafri et al.^24^ We based on our previous work and cafri's methodology^23^ to analysis the statistical power of relative risk of all AEs. The power analysis suggests that the power of RR of 0.99 for constipation was 5.03%, the power of RR of 0.90 for nausea was 7.24%, the power of RR of 0.89 for vomiting was 6.49%, the power of RR of 0.80 for pruritus was 6.34%, the power of RR of 0.62 for sleepiness was 12.82%, the power of RR of 0.94 for dizziness was 5.34%, the power of RR of 0.97 for anorexia was 5.02%, and the power of RR of 0.34 for dysuria was 8.78%,

### Publication Bias

The publication bias of our meta-analysis was assessed using funnel Begg and Egger regressions. Insufficient evidence of publication bias was found from the formal statistical tests (dysuria: Begg test, *P* = 1.00; Egger test, *P* = 0.41; constipation: Begg test, *P* = 0.64; Egger test, *P* = 0.78; nausea: Begg test, *P* = 0.06; Egger test, *P* = 0.06; vomiting: Begg test, *P* = 0.16; Egger test, *P* = 0.09; pruritus: Begg test, *P* = 0.73; Egger test, *P* = 0.43; sleepiness: Begg test, *P* = 1.00; Egger test, *P* = 0.34; dizziness: Begg test, *P* = 0.05; Egger test, *P* = 0.07; and anorexia: Begg test, *P* = 0.31; Egger test, *P* = 0.17) (Supplemental Data 4).

### Grades of Evidence

Eight side effects were included in our meta-analysis, and all of 8 outcomes expect pruritus were important results. GRADE Working Group levels of evidence were high for all of 8 AEs. Supplemental Data 5 showed the detail of the quality of the evidence.

## DISCUSSION

Oxycodone, a semi-synthetic opioid, stimulate the receptors of μ, κ, and δ, which is effective in cancer-related pain, and the efficacy of oxycodone has a relative ratio of 1/1.5 to 2.0 compared with morphine.^1^ Oxycodone was 1 of first-line oral opioids in the treatment of cancer-related pain,^27,28^ regardless of renal function,^29^ it was suggested as an effective alternative to oral opioids.^1,10,12,30^ The quality of life of cancer patients improved too with oxycodone treatment.^31^ Including China, the use of oxycodone had increased significantly in many countries.^30^ Similar to other opioids, nausea, constipation, dizziness, vomiting, sleepiness, pruritus, anorexia, and dysuria were the most common AEs,^12^ so it is critical to determine that if there were significantly different in AEs among oxycodone and other opioids when treated in cancer-related pain.

This systematic review and meta-analysis involved 10 studies and 11 trials, enrolled a total of 1211 patients. Current literature demonstrated that oxycodone had no significant difference in the RR of all AEs compared with other opioids (dizziness: RR = 0.94, 95% CI: 0.69–1.30, Z = 0.35, *P* = 0.72; nausea: RR = 0.88, 95% CI: 0.72–1.07, Z = 1.26, *P* = 0.21; vomiting: RR = 0.89, 95% CI: 0.70–1.15, Z = 0.9, *P* = 0.37; sleepiness: RR = 0.86, 95% CI: 0.38–1.36, Z = 0.36, *P* = 0.72; constipation: RR = 0.98, 95% CI: 0.81–1.19, Z = 0.21, *P* = 0.83; anorexia: RR = 0.97, 95% CI = 0.58–1.62, Z = 0.11, *P* = 0.91; pruritus: RR = 0.76, 95% CI: 0.44–1.30, Z = 1.01, *P* = 0.31; and dysuria: RR = 0.33, 95% CI: 0.07–1.62, Z = 1.36, *P* = 0.17). While subgroup analysis shows that there were significantly different between Ox CR (n = 12) and Mc CR (n = 25) in sleepiness (RR = 0.47, 95% CI: 0.25–0.90, *P* = 0.02).

The efficacy of oxycodone in the treatment of cancer-related pain had been proved to be superior to other opioids,^12^ and our meta-analysis demonstrated that the all AEs of oxycodone were similar to other opioids. Therefore, oxycodone could be a critical opioids in the management of moderate or severe pain in cancer patients in clinic. However, opioids were the second-line drugs in the management of neuropathic, tricyclic antidepressants were more appropriate compared with opioids.^32^

Our study has several strengths compared early meta-analysis.^12^ To our best knowledge, this meta-analysis was the first article based on the current evidence, which focus on the side effects of oxycodone in patients with cancer pain. Besides risk of bias was assessed based on Cochrane Collaboration tool, and the methodological quality of included studies were good and fair. In addition, power analysis for this meta-analysis was conducted. Finally, no significant publication bias about all AEs was found.

There were also some limitations in our meta-analysis. First, the statistical power of the RR of all AEs were low, so the results may not be sufficient convinced. Second, only small number of participates were included to evaluate the RRs of all AEs, and may reduce the power of our analysis; therefore, further studies should involve larger patients. Finally, not all types of cancers were involved, and the AEs of oxycodone may be different among various cancers originally.

In conclusion, this meta-analysis suggested that there were no statistically different among oxycodone and other opioids in all AEs, subgroup analysis showed that Ox CR may had less sleepiness compare with Mc CR. However, low power analysis not reaches power of test and insufficient patients were existed in our study; this conclusion should be interpreted cautiously. Therefore, further high-quality RCTs are warranted in this field.

## Supplementary Material

Supplemental Digital Content
