# Supplementary material for: The Adverse Events of Oxycodone in Cancer-Related Pain: A Systematic Review and Meta-Analysis of Randomized Controlled Trials
Source: Medicine (Baltimore). 2016 Apr 18;95(15):e3341. doi: 10.1097/MD.0000000000003341 (PMC4839832; doi:10.1097/MD.0000000000003341)
Supplement: Supplemental Digital Content [file medi-95-e3341-s001.pdf]

PRISMA 2009 Checklist

Section/Topic	#	Checklist Item	Reported on Page #
**TITLE**			
Title	1	Identify the report as a systematic review, meta-analysis, or both.	Title 1#
**ABSTRACT**			
Structured summary	2	Provide a structured summary including, as applicable: background; objectives; data sources; study eligibility criteria, participants, and interventions; study appraisal and synthesis methods; results; limitations; conclusions and implications of key findings; systematic review registration number.	Abstract 2-3#
**INTRODUCTION**			
Rationale	3	Describe the rationale for the review in the context of what is already known.	Introduction 3-4#
Objectives	4	Provide an explicit statement of questions being addressed with reference to participants, interventions, comparisons, outcomes, and study design (PICOS).	Introduction 3-4#
**METHODS**			
Protocol and registration	5	Indicate if a review protocol exists, if and where it can be accessed (e.g., Web address), and, if available, provide registration information including registration number.	Methods 4#
Eligibility criteria	6	Specify study characteristics (e.g., PICOS, length of follow-up) and report characteristics (e.g., years considered, language, publication status) used as criteria for eligibility, giving rationale.	Selection criteria and Data extraction 4-5#
Information sources	7	Describe all information sources (e.g., databases with dates of coverage, contact with study authors to identify additional studies) in the search and date last searched.	Search strategy 4#
Search	8	Present full electronic search strategy for at least one database, including any limits used, such that it could be repeated.	Appendix: PubMed and Embase

			search terms 11#
Study selection	9	State the process for selecting studies (i.e., screening, eligibility, included in systematic review, and, if applicable, included in the meta-analysis).	Selection criteria 4-5#
Data collection process	10	Describe method of data extraction from reports (e.g., piloted forms, independently, in duplicate) and any processes for obtaining and confirming data from investigators.	Data extraction 5#
Data items	11	List and define all variables for which data were sought (e.g., PICOS, funding sources) and any assumptions and simplifications made.	Selection criteria and Data extraction 4-5#
Risk of bias in individual studies	12	Describe methods used for assessing risk of bias of individual studies (including specification of whether this was done at the study or outcome level), and how this information is to be used in any data synthesis.	Assessment for Risk 5#
Summary measures	13	State the principal summary measures (e.g., risk ratio, difference in means).	Selection criteria and Data extraction 4-5#
Synthesis of results	14	Describe the methods of handling data and combining results of studies, if done, including measures of consistency (e.g., I^2) for each meta-analysis.	Statistical methods 5-6#

Section/Topic	#	Checklist Item	Reported on Page #
Risk of bias across studies	15	Specify any assessment of risk of bias that may affect the cumulative evidence (e.g., publication bias, selective reporting within studies).	Publication bias 9#

PRISMA 2009 Checklist

Additional analyses	16	Describe methods of additional analyses (e.g., sensitivity or subgroup analyses, meta-regression), if done, indicating which were pre-specified.	subgroup analyses 7-8#
**RESULTS**			
Study selection	17	Give numbers of studies screened, assessed for eligibility, and included in the review, with reasons for exclusions at each stage, ideally with a flow diagram.	Results 6#
Study characteristics	18	For each study, present characteristics for which data were extracted (e.g., study size, PICOS, follow-up period) and provide the citations.	Results 6#
Risk of bias within studies	19	Present data on risk of bias of each study and, if available, any outcome level assessment (see item 12).	Subgroups analysis 6-7#
Results of individual studies	20	For all outcomes considered (benefits or harms), present, for each study: (a) simple summary data for each intervention group (b) effect estimates and confidence intervals, ideally with a forest plot.	Relative risk for all AEs 7-8#
Synthesis of results	21	Present the main results of the review. If meta-analyses done, include for each, confidence intervals and measures of consistency.	Results 7-8#
Risk of bias across studies	22	Present results of any assessment of risk of bias across studies (see Item 15).	Publication bias 9#
Additional analysis	23	Give results of additional analyses, if done (e.g., sensitivity or subgroup analyses, meta-regression [see Item 16]).	subgroup analyses 7-8#
**DISCUSSION**			
Summary of evidence	24	Summarize the main findings including the strength of evidence for each main outcome; consider their relevance to key groups (e.g., healthcare providers, users, and policy makers).	Discussion 9-10#
Limitations	25	Discuss limitations at study and outcome level (e.g., risk of bias), and at review-level (e.g., incomplete retrieval of identified research, reporting bias).	Discussion 10#
Conclusions	26	Provide a general interpretation of the results in the context of other evidence, and implications for future research.	Discussion 10-11#
**FUNDING**			

PRISMA 2009 Checklist

Funding	27	Describe sources of funding for the systematic review and other support (e.g., supply of data); role of funders for the systematic review.	Funding 11#

From: Moher D, Liberati A, Tetzlaff J, Altman DG, The PRISMA Group (2009). Preferred Reporting Items for Systematic Reviews and Meta-Analyses: The PRISMA Statement. PLoS Med 6(6): e1000097. doi:10.1371/journal.pmed1000097

For more information, visit: **www.prisma-statement.org**.

The Adverse Events of Oxycodone in Cancer-Related Pain: A Systematic Review and Meta-Analysis of randomised controlled trials

Hu Ma MD, Ph.D, Yuan Liu MM, Su-Han Jin MM, Xian-Tao Zeng Ph.D, Joey S.W.Kwong Ph.D, Yu-Ju Bai MD, Xu Tian MN, RN, Jian-Guo Zhou MD*

Supplemental Data 2. The Search Strategy of PubMed and Embase

PubMed Search terms

#1 Search ("Oxycodone"[Mesh]) OR ((((((((((Oxycone[Title/Abstract]) OR Dinarkon[Title/Abstract]) OR Oxycodeinon[Title/Abstract]) OR Dihydrohydroxycodeinone[Title/Abstract]) OR Dihydrone[Title/Abstract]) OR Oxiconum[Title/Abstract]) OR Theocodin[Title/Abstract]) OR Oxycontin[Title/Abstract]) OR Purdue Frederick Brand of Oxycodone[Title/Abstract]) OR Pancodine[Title/Abstract]) OR Eucodal[Title/Abstract]) OR Oxycodone Hydrochloride [Title/Abstract])

#2 Search (cancer[MeSH Terms]) OR (((((((((Neoplasm[Title/Abstract]) OR Tumors[Title/Abstract]) OR Tumor[Title/Abstract]) OR Neoplasia[Title/Abstract]) OR Benign Neoplasms[Title/Abstract]) OR Neoplasms, Benign[Title/Abstract]) OR Benign Neoplasm[Title/Abstract]) OR Neoplasm, Benign[Title/Abstract]) OR Cancer[Title/Abstract]) OR Cancers[Title/Abstract])

#3 Search (((pain[Title/Abstract]) OR Pain Measurement[MeSH Major Topic]) OR Pain Management[MeSH Major Topic]) OR pain[MeSH Terms]

#4 Search ((("Controlled Clinical Trial"[Publication Type]) OR ("Randomized Controlled Trials as Topic"[Mesh] OR "Randomized Controlled Trial"[Publication Type] OR "Controlled Clinical Trials as Topic"[Mesh]))) OR ((((((Controlled Clinical Trial[Title/Abstract]) OR Controlled Clinical Trials, Randomized[Title/Abstract]) OR Clinical Trials, Randomized[Title/Abstract]) OR Trials, Randomized Clinical[Title/Abstract]) OR Controlled Clinical Trials[Title/Abstract]) OR random*[Title/Abstract])

#5 #1 AND #2 AND #3 AND #4

Embase Search terms

#1 ‘oxycodone’ OR ‘Oxycone’ OR ‘Dinarkon’ OR ‘Oxycodeinon’ OR ‘Oxycodone Hydrochloride’ OR ‘Dihydrone’ OR ‘Oxiconum’ OR ‘Theocodin’ OR ‘Oxycontin’ OR ‘Purdue Frederick Brand of Oxycodone’ OR ‘Pancodine’ OR ‘Eucodal’ OR ‘Oxycodone Hydrochloride’

#2 'controlled clinical trial'/exp OR 'controlled clinical trial' OR 'randomized controlled trials'/exp OR 'randomized controlled trials' OR 'controlled clinical trials'/exp OR 'controlled clinical trials' OR 'controlled clinical trials, randomized' OR 'clinical trials, randomized' OR 'trials, randomized clinical' OR 'random'

#3 ‘Pain’ OR ‘Pain Management’ OR ‘Pain Measurement’

#4 ‘cancer’ OR ‘Neoplasm’ OR ‘Tumors’ OR ‘Tumor’ OR ‘Neoplasia’ OR ‘Cancer’ OR ‘Cancers’

#5 #1 AND #2 AND #3 AND #4

The Adverse Events of Oxycodone in Cancer-Related Pain: A Systematic Review and Meta-Analysis of randomised controlled trials

Hu Ma MD, Ph.D, Yuan Liu MM, Su-Han Jin MM, Xian-Tao Zeng Ph.D, Joey S.W.Kwong Ph.D, Yu-Ju Bai MD, Xu Tian MN, RN, Jian-Guo Zhou MD*

Supplemental Data 2. The Result of SAS Meta Power Anlysis

```
data constipation;
input es v;
cards;
-2.1776161	2.2026125
0.04546237	0.03357327
0.64435702	0.46746032
0.37320425	0.38440021
0	0.31292517
-0.06499214	0.0651521
0.06669137	0.16662959
0.15822401	0.08037166
-0.24116206	0.2474026
-0.48550782	0.12019231
0.54654371	0.65191388
;
run;
%metapower (test='M', model='fixed', raw_data='yes', alpha=.05, tau2=99,heterogeneity=99, n1=99, n2=99, k=99, eff_type='or', T= -0.005243, Dataset= constipation, B=NA, v=v, x=NA, es=es, p=NA, weight=NA);
run;
```

Output - (Untitled)

```
                    The SAS System          2015年10月21日 星期三 上午04时57分10秒

        --------------------Meta-Analysis Power Macro--------------------

                          Test of Mean Effect Size

                         Model =             fixed
                         Effect Size Metric = or

                           Raw data provided= Yes

                        Mean Effect Size =  -0.005243
                        Number of Studies =        11
                        Sampling Variance = 0.0116463
                        Alpha =                  0.05

              Estimated Power of Test (One-Tailed) = 0.0451862
```

Estimated Power of Test (Two-Tailed) = 0.0502704

```
data Nausea;
input es v;
cards;
-0.78249228   0.34214993
0.04546237    0.03357327
-0.25299651   0.12898099
-0.19798988   0.13354727
0.48972545    0.20647408
-0.08742281   0.08774091
-0.18232156   0.25648148
-0.08590334   0.08628251
-0.63252256   1.3786765
-0.19415601   0.39201681
-1.1631508    0.535
;
run;
%metapower (test='M', model='fixed', raw_data='yes', alpha=.05, tau2=99,heterogeneity=99,
n1=99, n2=99, k=99, eff_type='or', T= -0.04769, Dataset= Nausea, B=NA, v=v, x=NA, es=es, p=NA,
weight=NA);
run;
```

The SAS System 2015年10月21日 星期三 上午05时00分50秒 1

---------------------Meta-Analysis Power Macro--------------------

Test of Mean Effect Size

Model = fixed

Effect Size Metric = or

Raw data provided= Yes

Mean Effect Size = -0.04769

Number of Studies = 11

Sampling Variance = 0.0117891

Alpha = 0.05

Estimated Power of Test (One-Tailed) = 0.0185765

Estimated Power of Test (Two-Tailed) = 0.0723783

```
data Vomiting;
input es v;
cards;
-2.5261095    2.1344214
0.07711303    0.03269768
-0.61558895   0.26894559
-0.16632122   0.33466462
-0.17206506   0.34615331
0.28995222    0.29632035
0.23638878    0.33060429
```

```
-0.54796517   0.24172297
-0.34830669   0.71895425
-0.25782911   0.52361854
0.30538165    0.73308271;
run;
%metapower (test='M', model='fixed', raw_data='yes', alpha=.05, tau2=99,heterogeneity=99,
n1=99, n2=99, k=99, eff_type='or', T= -0.04866, Dataset= Vomiting, B=NA, v=v, x=NA, es=es,
p=NA, weight=NA);
run;
```


---------------------Meta-Analysis Power Macro---------------------

Test of Mean Effect Size

Model = fixed

Effect Size Metric = or

Raw data provided= Yes

Mean Effect Size =	-0.04866
Number of Studies =	10
Sampling Variance =	0.018354
Alpha =	0.05

Estimated Power of Test (One-Tailed) = 0.0225335

Estimated Power of Test (Two-Tailed) = 0.0649056

```
data Pruritus;
input es v;
cards;
-0.3801473     0.15970085
-0.131336      0.41322537
-0.34830669    0.71895425
0.19237189     0.48787879
;
run;
%metapower (test='M', model='fixed', raw_data='yes', alpha=.05, tau2=99,heterogeneity=99,
n1=99, n2=99, k=99, eff_type='or', T= −0.097997, Dataset= Pruritus, B=NA, v=v, x=NA, es=es,
p=NA, weight=NA);
run;
```

Estimated Power of Test (Two-Tailed) = 0.06344

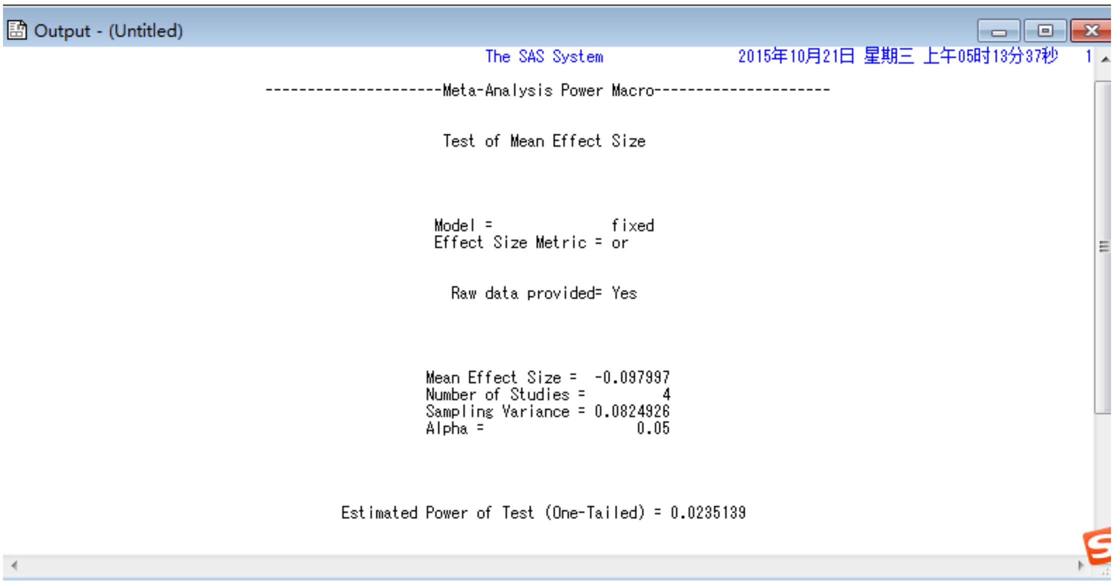

```
Output - (Untitled)
                         The SAS System          2015年10月21日 星期三 上午05时13分37秒   1

           --------------------Meta-Analysis Power Macro--------------------

                              Test of Mean Effect Size


                              Model =                fixed
                              Effect Size Metric = or

                                Raw data provided= Yes


                            Mean Effect Size =   -0.097997
                            Number of Studies =          4
                            Sampling Variance = 0.0824926
                            Alpha =                   0.05


                  Estimated Power of Test (One-Tailed) = 0.0235139
```

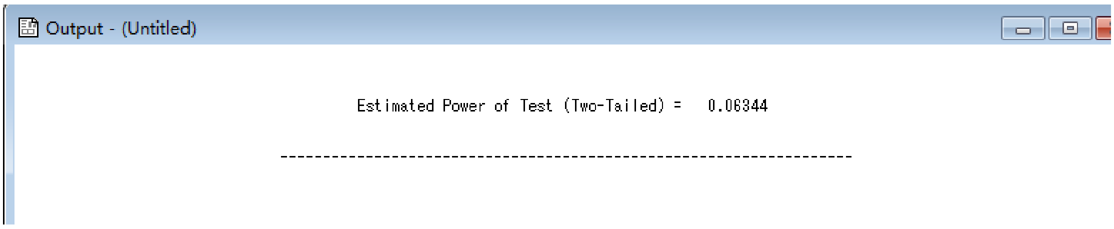

```
Output - (Untitled)

                  Estimated Power of Test (Two-Tailed) =   0.06344

          -----------------------------------------------------------------
```

```
data Sleepiness;
input es v;
cards;
-0.78495473    0.15982531
-0.23687374    0.20854629
0      0.88235294
-0.63252256    0.68933824
-0.04879016    0.56904762
;
run;
%metapower (test='M', model='fixed', raw_data='yes', alpha=.05, tau2=99,heterogeneity=99,
n1=99, n2=99, k=99, eff_type='or', T= -0.20691, Dataset= Sleepiness, B=NA, v=v, x=NA, es=es,
p=NA, weight=NA);
run;
```

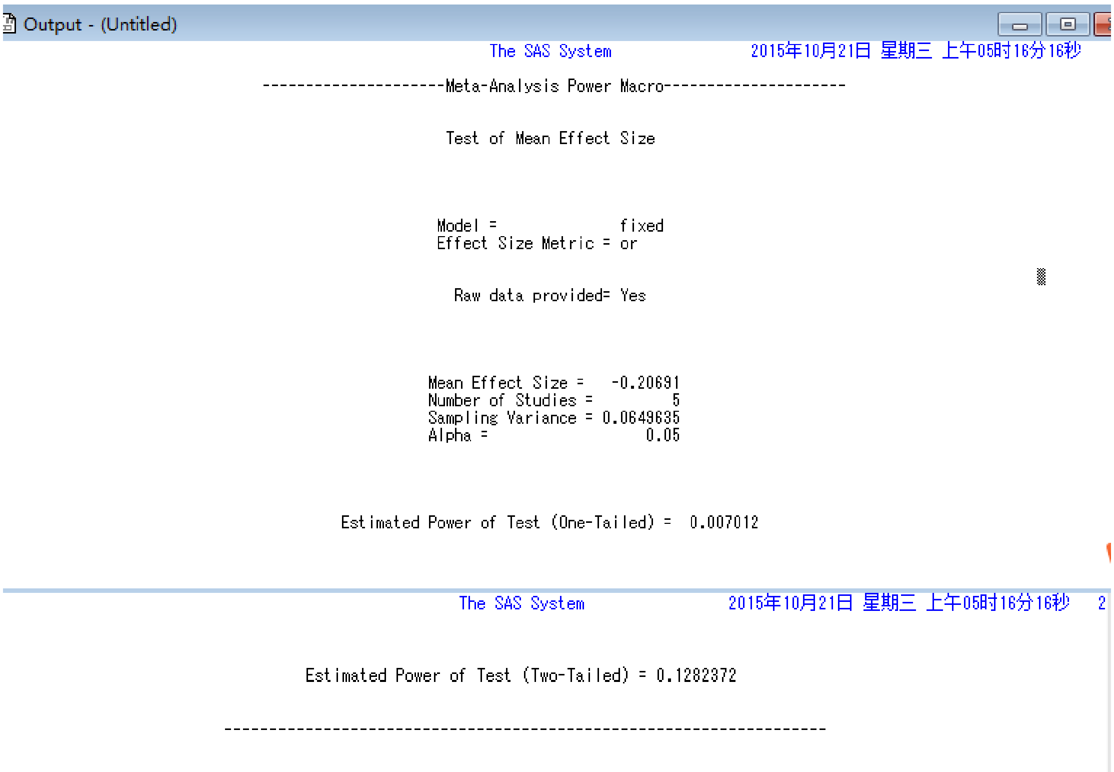

```
Output - (Untitled)
                              The SAS System          2015年10月21日 星期三 上午05时16分16秒

                 --------------------Meta-Analysis Power Macro--------------------

                                Test of Mean Effect Size


                                Model =              fixed
                                Effect Size Metric = or


                                 Raw data provided= Yes


                               Mean Effect Size =    -0.20691
                               Number of Studies =          5
                               Sampling Variance = 0.0649635
                               Alpha =                   0.05


                        Estimated Power of Test (One-Tailed) =  0.007012

                              The SAS System          2015年10月21日 星期三 上午05时16分16秒   2

                     Estimated Power of Test (Two-Tailed) = 0.1282372

           -----------------------------------------------------------------
```

```
data dizziness;
input es v;
cards;
-0.55004634   0.37371795
0.05339289    0.07932773
-0.38865799   0.79971751
-0.65981076   0.7166574
0.20686267    0.20950185
-0.43332206   0.35667722
0      0.27272727
0.17185026    0.34736842
-0.66139848   1.4364919
0.27193372    0.32738095
;
run;
%metapower (test='M', model='fixed', raw_data='yes', alpha=.05, tau2=99,heterogeneity=99,
n1=99, n2=99, k=99, eff_type='or', T= -0.02872, Dataset= dizziness, B=NA, v=v, x=NA, es=es, p=NA,
weight=NA);
run;
```

Output - (Untitled)

```
                                  The SAS System            2015年10月21日 星期三 上午05时20分30秒   1

                --------------------Meta-Analysis Power Macro---------------------

                                Test of Mean Effect Size


                               Model =             fixed
                               Effect Size Metric = or


                                Raw data provided= Yes



                              Mean Effect Size =   -0.02872
                              Number of Studies =        10
                              Sampling Variance = 0.0279325
                              Alpha =                  0.05


                      Estimated Power of Test (One-Tailed) = 0.0346319
```

Output - (Untitled)

```
                      Estimated Power of Test (Two-Tailed) = 0.0533896

                ------------------------------------------------------------------
```

```
data anorexia;
input es v;
cards;
0.05571061    0.08377805
-1.063521     2.5964696
-0.34830669   0.71895425
-0.05406722   1.8918129
;
run;
%metapower (test='M', model='fixed', raw_data='yes', alpha=.05, tau2=99,heterogeneity=99,
n1=99, n2=99, k=99, eff_type='or', T= -0.01278, Dataset= anorexia, B=NA, v=v, x=NA, es=es, p=NA,
weight=NA);
run;
```

Output - (Untitled)

```
                              The SAS System              2015年10月21日 星期三 上午05时22分06秒   1

                  --------------------Meta-Analysis Power Macro--------------------

                                   Test of Mean Effect Size


                                Model =               fixed
                                Effect Size Metric =  or


                                  Raw data provided= Yes



                               Mean Effect Size =    -0.01278
                               Number of Studies =          4
                               Sampling Variance =  0.0702201
                               Alpha =                   0.05


                         Estimated Power of Test (One-Tailed) =    0.04522
```

put - (Untitled)

```
                    Estimated Power of Test (Two-Tailed) = 0.0502665

             ----------------------------------------------------------------------
```

```
data Dysuia;
input es v;
cards;
-1.5755364     2.366092
-1.0815179     2.6324761
-0.66139848    1.4364919
;
run;
%metapower (test='M', model='fixed', raw_data='yes', alpha=.05, tau2=99,heterogeneity=99,
n1=99, n2=99, k=99, eff_type='or', T= -0.464705879957229, Dataset= Dysuia, B=NA, v=v, x=NA,
es=es, p=NA, weight=NA);
run;
```

Output - (Untitled)

```
                         The SAS System               2015年10月21日 星期三 上午05时23分12秒   1

    --------------------Meta-Analysis Power Macro--------------------

                         Test of Mean Effect Size



                        Model =               fixed
                        Effect Size Metric = or


                          Raw data provided= Yes



                       Mean Effect Size =   -0.464706
                       Number of Studies =          3
                       Sampling Variance = 0.6672678
                       Alpha =                   0.05



             Estimated Power of Test (One-Tailed) = 0.0134232
```

Output - (Untitled)

```
                         The SAS System               2015年10月21日 星期三 上午05时23分12秒   2


             Estimated Power of Test (Two-Tailed) = 0.0878232

    ------------------------------------------------------------------
```

The Adverse Events of Oxycodone in Cancer-Related Pain: A Systematic Review and Meta-Analysis of randomised controlled trials

Hu Ma MD, Ph.D, Yuan Liu MM, Su-Han Jin MM, Xian-Tao Zeng Ph.D, Joey S.W.Kwong Ph.D, Yu-Ju Bai MD, Xu Tian MN, RN, Jian-Guo Zhou MD*

Supplemental Data 4. The Result of Publication Bias by Egger' and Begg' Regression

The publication bias of our meta-analysis was assessed using funnel Begg's and Egger's regreesion. There was no evidence of significant publication bias by inspection of the formal statistical tests [(1) dysuria: Begg's test, *P* = 1.00; Egger's test, *P* = 0.41); (2) constipation: Begg's test, *P* = 0.64; Egger's test, *P* = 0.78); nausea: Begg's test, *P* = 0.06; Egger's test, *P* = 0.06); vomiting: Begg's test, *P* = 0.16; Egger's test, *P* = 0.09); pruritus: Begg's test, *P* = 0.73; Egger's test, *P* = 0.43); sleepiness: Begg's test, *P* = 1.00; Egger's test, *P* = 0.34); dizziness: Begg's test, *P* = 0.05; Egger's test, *P* = 0.07); anorexia: Begg's test, *P* = 0.31; Egger's test, *P* = 0.17)].

1. Dysuria:

. metabias logrr _selogES, _ES egger

Note: default data input format (theta, se_theta) assumed.

Tests for Publication Bias

Begg's Test

```
   adj. Kendall's Score (P-Q) =         -1
           Std. Dev. of Score =       1.91
            Number of Studies =          3
                          z   =      -0.52
                    Pr > |z| =      0.602
                          z   =       0.00 (continuity corrected)
                    Pr > |z| =      1.000 (continuity corrected)
```

Egger's test

```
------------------------------------------------------------------------------
     Std_Eff |      Coef.   Std. Err.      t    P>|t|     [95% Conf. Interval]
```

slope	1.210149	1.716098	0.71	0.609	-20.59495	23.01525
bias	-1.594985	1.212918	-1.31	**0.414**	-17.00657	13.8166

2. Constipation

Note: default data input format (theta, se_theta) assumed.

Tests for Publication Bias

Begg's Test

adj. Kendall's Score (P-Q) = 7
Std. Dev. of Score = 12.85
Number of Studies = 11
z = 0.54
Pr > |z| = 0.586
z = 0.47 (continuity corrected)
Pr > |z| = 0.640 (continuity corrected)

Egger's test

Std_Eff	Coef.	Std. Err.	t	P>|t|	[95% Conf. Interval]	
slope	.0478924	.19199	0.25	0.809	-.3864192	.4822041
bias	-.1554965	.5363993	-0.29	**0.778**	-1.368916	1.057923

3. Nausea

Note: default data input format (theta, se_theta) assumed.

Tests for Publication Bias

Begg's Test

adj. Kendall's Score (P-Q) = -25
Std. Dev. of Score = 12.85
Number of Studies = 11
z = -1.95
Pr > |z| = 0.052
z = 1.87 (continuity corrected)
Pr > |z| = 0.062 (continuity corrected)

Egger's test

Std_Eff	Coef.	Std. Err.	t	P>|t|	[95% Conf. Interval]	
slope	.2309468	.1691424	1.37	0.205	-.1516799	.6135735
bias	-.9987682	.4696943	-2.13	**0.062**	-2.061291	.0637542

----

4. Vomiting

Note: default data input format (theta, se_theta) assumed.

Tests for Publication Bias

Begg's Test

adj. Kendall's Score (P-Q) = -19
Std. Dev. of Score = 12.85
Number of Studies = 11
z = -1.48
Pr > |z| = 0.139
z = 1.40 (continuity corrected)
Pr > |z| = 0.161 (continuity corrected)

Egger's test

Std_Eff	Coef.	Std. Err.	t	P>|t|	[95% Conf. Interval]	
slope	.2297391	.1821207	1.26	0.239	-.1822466	.6417248
bias	-.7686158	.4103623	-1.87	**0.094**	-1.69692	.1596883

5. Pruritus

.

Note: default data input format (theta, se_theta) assumed.

Tests for Publication Bias

Begg's Test

adj. Kendall's Score (P-Q) = 2
Std. Dev. of Score = 2.94
Number of Studies = 4
z = 0.68
Pr > |z| = 0.497
z = 0.34 (continuity corrected)

Pr > |z| = 0.734 (continuity corrected)

Egger's test

Std_Eff	Coef.	Std. Err.	t	P>|t|	[95% Conf. Interval]	
slope	-.6339154	.4302076	-1.47	0.279	-2.484949	1.217119
bias	.7341634	.7489287	0.98	**0.430**	-2.488217	3.956544

6. Sleepiness

Note: default data input format (theta, se_theta) assumed.

Tests for Publication Bias

Begg's Test

adj. Kendall's Score (P-Q) = 0
Std. Dev. of Score = 4.08
Number of Studies = 5
z = 0.00
Pr > |z| = 1.000
z = -0.24 (continuity corrected)
Pr > |z| = 1.000 (continuity corrected)

Egger's test

Std_Eff	Coef.	Std. Err.	t	P>|t|	[95% Conf. Interval]	
slope	-.9429237	.4569466	-2.06	0.131	-2.397132	.5112842
bias	.9008406	.8017623	1.12	**0.343**	-1.650725	3.452406

7. Dizziness

Note: default data input format (theta, se_theta) assumed.

Tests for Publication Bias

Begg's Test

adj. Kendall's Score (P-Q) = -23
Std. Dev. of Score = 11.18
Number of Studies = 10
z = -2.06

Pr > |z| = 0.040
z = 1.97 (continuity corrected)
Pr > |z| = 0.049 (continuity corrected)

Egger's test

Std_Eff	Coef.	Std. Err.	t	P>|t|	[95% Conf. Interval]	
slope	.3788555	.2154277	1.76	0.117	-.1179216	.8756325
bias	-.8627398	.4076116	-2.12	**0.067**	-1.802694	.0772142

8. Anorexia

Note: default data input format (theta, se_theta) assumed.

Tests for Publication Bias

Begg's Test

adj. Kendall's Score (P-Q) = -4
Std. Dev. of Score = 2.94
Number of Studies = 4
z = -1.36
Pr > |z| = 0.174
z = 1.02 (continuity corrected)
Pr > |z| = 0.308 (continuity corrected)

Egger's test

Std_Eff	Coef.	Std. Err.	t	P>|t|	[95% Conf. Interval]	
slope	.2105003	.1366696	1.54	0.263	-.3775416	.7985421
bias	-.5442062	.2578762	-2.11	**0.169**	-1.653758	.5653456

The Adverse Events of Oxycodone in Cancer-Related Pain: A Systematic Review and Meta-Analysis of randomised controlled trials

Hu Ma MD, Ph.D, Yuan Liu MM, Su-Han Jin MM, Xian-Tao Zeng Ph.D, Joey S.W.Kwong Ph.D, Yu-Ju Bai MD, Xu Tian MN, RN, Jian-Guo Zhou MD*

Supplemental Data 5 The GRADE profile evidence of the included studies

Date: 2015-10-24

Question: Should Oxycodone vs Other opioid agents be used in Cancer-Related Pain?

Settings: The Adverse Events of Oxycodone in Cancer-Related Pain

Bibliography:

Quality assessment							No of patients		Effect		Quality	Importance
No of studies	Design	Risk of bias	Inconsistency	Indirectness	Imprecision	Other considerations	Oxycodone	Other opioid agents	Relative (95% CI)	Absolute		

**Dysuria**												
3	randomised trials	no serious risk of bias	no serious inconsistency	no serious indirectness	no serious imprecision	none	1/144 (0.69%)	5/144 (3.5%)	RR 0.343 (0.071 to 1.667)	23 fewer per 1000 (from 32 fewer to 23 more)	⊕⊕⊕⊕ HIGH	IMPORTANT
								0%		-		
**Constipation**												
11	randomised trials	no serious risk of bias	no serious inconsistency	no serious indirectness	no serious imprecision	none	128/604 (21.2%)	132/607 (21.7%)	RR 0.988 (0.800 to 1.220)	3 fewer per 1000 (from 43 fewer to 48 more)	⊕⊕⊕⊕ HIGH	IMPORTANT
								0%		-		
**Anorexia**												
4	randomised trials	no serious risk of bias	no serious inconsistency	no serious indirectness	no serious imprecision	none	24/183 (13.1%)	25/184 (13.6%)	RR 0.971 (0.579 to 1.628)	4 fewer per 1000 (from 57 fewer to 85 more)	⊕⊕⊕⊕ HIGH	IMPORTANT
								0%		-		
**Pruritus**												
4	randomised trials	no serious risk of bias	no serious inconsistency	no serious indirectness	no serious imprecision	none	18/118 (15.3%)	24/121 (19.8%)	RR 0.798 (0.456 to 1.398)	40 fewer per 1000 (from 108 fewer to 79 more)	⊕⊕⊕⊕ HIGH	NOT IMPORTANT
								0%		-		
**Sleepiness**												
5	randomised trials	no serious risk of bias	no serious inconsistency	no serious indirectness	no serious imprecision	none	22/211 (10.4%)	38/212 (17.9%)	RR 0.621 (0.379 to 1.018)	68 fewer per 1000 (from 111 fewer to 3 more)	⊕⊕⊕⊕ HIGH	IMPORTANT
								20%		76 fewer per 1000 (from 124 fewer to 4		

										more)		
**Vomiting**												
11	randomised trials	no serious risk of bias	no serious inconsistency	no serious indirectness	no serious imprecision	none	88/604 (14.6%)	100/607 (16.5%)	RR 0 (0.690 to 1.158)	165 fewer per 1000 (from 51 fewer to 26 more)	⊕⊕⊕⊕ HIGH	IMPORTANT
								0%		-		
**Nausea**												
11	randomised trials	no serious risk of bias	no serious inconsistency	no serious indirectness	no serious imprecision	none	125/604 (20.7%)	144/607 (23.7%)	RR 0.896 (0.725 to 1.108)	25 fewer per 1000 (from 65 fewer to 26 more)	⊕⊕⊕⊕ HIGH	IMPORTANT
								0%		-		
**Dizziness**												
10	randomised trials	no serious risk of bias	no serious inconsistency	no serious indirectness	no serious imprecision	none	61/512 (11.9%)	65/515 (12.6%)	RR 0.936 (0.676 to 1.295)	8 fewer per 1000 (from 41 fewer to 37 more)	⊕⊕⊕⊕ HIGH	IMPORTANT
								0%		-		
